# 101 contact twins in gypsum experimentally obtained from calcium carbonate enriched solutions: mineralogical implications for natural gypsum deposits

**DOI:** 10.1107/S1600576723002674

**Published:** 2023-04-13

**Authors:** Andrea Cotellucci, Fermín Otálora, Àngels Canals, Joaquin Criado-Reyes, Luca Pellegrino, Marco Bruno, Dino Aquilano, Juan Manuel Garcia-Ruiz, Francesco Dela Pierre, Linda Pastero

**Affiliations:** a Dipartimento di Scienze della Terra, Università degli Studi di Torino, Via Valperga Caluso 35, Torino 10125, Italy; b Instituto Andaluz de Ciencias de la Tierra, CSIC-UGR, Avda De las Palmeras 4, Granada, Armilla 18100, Spain; c Departament de Mineralogía, Petrología i Geología Aplicada, Facultad de Ciencias de la Terra, Universidad de Barcelona, Martí i Franqués 1, Barcelona 08028, Spain; Oak Ridge National Laboratory, USA; North Carolina State University, USA

**Keywords:** gypsum, twins, fluid inclusions, evaporites, swallowtail

## Abstract

Gypsum twins are frequently observed in nature, triggered by a wide array of impurities that are present in their depositional environments and that may exert a critical role in the selection of different twin laws. Identifying the impurities able to promote the selection of specific twin laws has relevance for geological studies aimed at interpreting the gypsum depositional environments in ancient and modern deposits. Here, the effect of calcium carbonate (CaCO_3_) on gypsum (CaSO_4_·2H_2_O) growth morphology has been investigated by performing temperature-controlled laboratory experiments with and without the addition of carbonate ions. The precipitation of twinned gypsum crystals has been achieved experimentally (101 contact twin law) by adding carbonate to the solution, and the involvement of rapidcreekite (Ca_2_SO_4_CO_3_·4H_2_O) in selecting the 101 gypsum contact twin law was supported, suggesting an epitaxial mechanism. Moreover, the occurrence of 101 gypsum contact twins in nature has been suggested by comparing the natural gypsum twin morphologies observed in evaporitic environments with those obtained in experiments. Finally, both orientations of the primary fluid inclusions (of the negative crystal shape) with respect to the twin plane and the main elongation of sub-crystals that form the twin are proposed as a fast and useful method (especially in geological samples) to distinguish between the 100 and 101 twin laws. The results of this study provide new insights into the mineralogical implications of twinned gypsum crystals and their potential as a tool to better understand natural gypsum deposits.

## Abstract 2

Identifying the impurities that are able to promote the selection of specific gypsum twin laws has relevance for geological studies aimed at interpreting the gypsum depositional environments in ancient and modern deposits. The results of this study provide new insights into the mineralogical implications of twinned gypsum crystals and should help future research to make a better use of the twin laws observed in gypsum in ancient sedimentary successions as a proxy for the chemistry of the original brine.

## 1. Introduction

Gypsum (calcium sulfate dihydrate, CaSO_4_·2H_2_O) is the most abundant natural sulfate mineral on Earth’s surface (Aquilano *et al.*, 2016 ▸) and is mostly found in evaporitic environments (*e.g.* Warren, 1982 ▸; Manzi *et al.*, 2009 ▸; Ortí, 2011 ▸; Lugli *et al.*, 2010 ▸; Van Driessche *et al.*, 2019 ▸; Costanzo *et al.*, 2019 ▸; Otálora *et al.*, 2020 ▸). Remarkably, the history of Earth, from the Neoproterozoic to the Phanerozoic, is punctuated by dramatic episodes of evaporitic deposition which resulted in the accumulation of thick gypsum or anhydrite-bearing sedimentary successions (*e.g.* Warren, 2010 ▸). Furthermore, gypsum deposits were also detected on Mars (Gendrin *et al.*, 2005 ▸; Langevin *et al.*, 2005 ▸), and a swallowtail gypsum habit, commonly referred to gypsum twins (Cody & Cody, 1989*b* ▸), was recently observed by the NASA Curiosity Mars rover (Edgar *et al.*, 2018 ▸).

Depending on the depositional environment, gypsum exhibits different habits. In soils (Jafarzadeh & Burnham, 1992 ▸), desert regions (Shahid & Abdelfattah, 2009 ▸) and salt lakes (Warren, 1982 ▸; Mees *et al.*, 2012 ▸), tabular, prismatic, acicular, lenticular and twinned crystals are observed. In marine evaporites, mostly twinned and tabular gypsum crystals are found (Ortí, 2011 ▸), whereas the most spectacular habits are related to the anhydrite–gypsum thermally driven transformation by a self-feeding mechanism in low supersaturated solutions. This latter condition induces the precipitation of the prismatic metre-sized single crystals and twins of the Naica Mine (Mexico) (García-Ruiz *et al.*, 2007 ▸; Otálora & García-Ruiz, 2014 ▸) and in the Geode of Pulpì (Almería, Spain) (Canals *et al.*, 2019 ▸).

Many crystal-growth experiments have been performed to establish which conditions favor particular gypsum habits. Acicular gypsum single crystals were observed (i) in a gel medium (Rinaudo *et al.*, 1985 ▸), (ii) from the hydration of bassanite (Craker & Schiller, 1962 ▸) and (iii) by evaporation of Ca^2+^–SO_4_^2−^ rich water solutions at 35°C (Montagnino *et al.*, 2011 ▸), to name a few. Reiss *et al.* (2019 ▸) observed that saline waters with Na^+^, K^+^, Mg^2+^, Sr^2+^, Cl^−^ and Br^−^ ions in solution reduce the [001] elongation of gypsum single crystals, which switch from the acicular to the tabular habit, whereas organic molecules from the decomposition of green plants promote the lenticular habit (Cody, 1979 ▸). Regarding gypsum twins, both 100 and 101 penetration twin laws of gypsum are common in pure aqueous solutions (Kern & Rehn, 1960 ▸), whereas α-amylase triggers the precipitation of a peculiar gypsum twin habit similar to those present in the Eocene deposits of the Paris Basin (Cody & Cody, 1989*a* ▸; Van Driessche *et al.*, 2019 ▸).

An epitaxial relationship between the {010} pinacoid of gypsum and the {10.4} rhombohedron of calcite (Ruiz-Agudo *et al.*, 2016 ▸; Aquilano *et al.*, 2022 ▸) has been demonstrated. Therefore, the effect of carbonate anions – ubiquitous in evaporitic environments – on the habit of gypsum crystals deserves further investigation. Consequently, the main objective of this work is to explore the effect of calcium carbonate (Ca-carbonate, hereinafter) on the gypsum habit by performing temperature-controlled laboratory experiments from aqueous solutions with Ca-carbonate added and not added. Our results indicate that an aqueous solution saturated in Ca-carbonate promotes the precipitation of twinned gypsum following the 101 contact twin law. To our knowledge, this is the first evidence of the effect of Ca-carbonate as a specific impurity in promoting the formation of the 101 contact twin law.

Rapidcreekite was detected as a precursor in gypsum precipitation when carbonate species are dissolved in solution (Bots, 2011 ▸). The structural affinities between these two minerals suggest that rapidcreekite could act as a precursor of gypsum via an epitaxial mechanism, promoting the formation of the 101 gypsum contact twin law.

Finally, we propose that the orientations of the negative crystal shape of primary fluid inclusions (FIs) with respect to the twin plane and the main direction of elongation of the sub-crystals that make up the twin are a useful tool to distinguish between 100 and 101 twins, which can be relevant for the interpretation of ancient gypsum deposits.

## 2. Materials and methods

CaSO_4_·2H_2_O reagent plus (≥99% Sigma–Aldrich), CaCO_3_ ACS reagent (≥99% powder, Sigma–Aldrich) and ultrapure water (18 MΩ, obtained using an Elga Purelab Flex3 water purification system) were used to prepare (i) CaSO_4_·2H_2_O saturated solution (G1) and (ii) CaSO_4_·2H_2_O–CaCO_3_ saturated solution (G2). Both G1 and G2 were saturated at 40°C. A cryo-compact Julabo circulator (CF31 series) was used to keep the solution at 40°C. Solubility values of CaSO_4_·2H_2_O at temperatures of 40 and 4°C were calculated using *PHREEQC* (version 3.7.3; Parkhurst & Appelo, 2013 ▸) and the default phreeqc database.

G1 was prepared by adding solid CaSO_4_·2H_2_O in amounts exceeding the saturation in pure water at 40°C (*i.e.* 2.66 g l^−1^). G2 was prepared by adding CaSO_4_·2H_2_O in amounts exceeding its saturation in pure water at 40°C to a solution already saturated with Ca-carbonate. Under these conditions, the surplus of CaCO_3_ and CaSO_4_·2H_2_O was stirred continuously in the flask for 15 days to ensure that saturation had been reached.

The pHs of G1 and G2 were 5.6 and 7.8, respectively. The pH of G2 is higher than that of G1 due to the basic hydrolysis of carbonate ions. pH measurements were carried out with a HANNA HI211 pH-meter.

Before the crystallization experiments, G1 and G2 were vacuum filtered in a beaker pre-heated to 40°C – to avoid rapid crystal precipitation – using a cellulose filter with a 0.45 µm pore size to remove any pre-existent CaCO_3_ and CaSO_4_·2H_2_O particles.

Volumes of 100 ml of G1 and G2 were placed in a refrigerator set at 4°C for 30 days in closed flasks to avoid evaporation. Gypsum precipitation was achieved through different CaSO_4_·2H_2_O–CaCO_3_ solubilities as a function of temperature. Lowering the temperature from 40 to 4°C caused a decrease in gypsum solubility – from 2.66 to 2.29 g l^−1^ – and an increase in CaCO_3_ solubility (Plummer & Busenberg, 1982 ▸; Coto *et al.*, 2012 ▸). Thus, Ca-carbonate minerals do not precipitate while both nucleation and growth of gypsum crystals occur.

Crystals were washed with ultrapure water, dried overnight at room temperature, and then analyzed by optical (Olympus BX4 with JENOPTIC ProgResC5 digital camera) and electron microscopes (JEOL JSM-IT300LV), equipped with a secondary, backscattered electron and energy-dispersive X-ray spectrometer.

Historically, gypsum was indexed in a variety of near-equivalent ways (*e.g.* Cole & Lancucki, 1974 ▸; Pedersen & Semmingsen, 1982 ▸; Comodi *et al.*, 2008 ▸). A comparison of these indexing possibilities is presented by Aquilano *et al.* (2016 ▸). Here, we adopted the monoclinic *C*2/*c* space group where *a*_0_ = 5.63 Å, *b*_0_ = 15.15 Å, *c*_0_ = 6.23 Å, α = γ = 90°, β = 113.50° (De Jong & Bouman, 1939 ▸). Our choice was based on two practical reasons: (i) this frame uses the smallest lattice vectors, and (ii) the [001] *z* axis coincides with the morphological elongation of the crystals growing from pure aqueous solution and the majority of natural crystals.

## 3. Results and discussions

### 3.1. Gypsum twin growth morphologies

**Figure 1:**
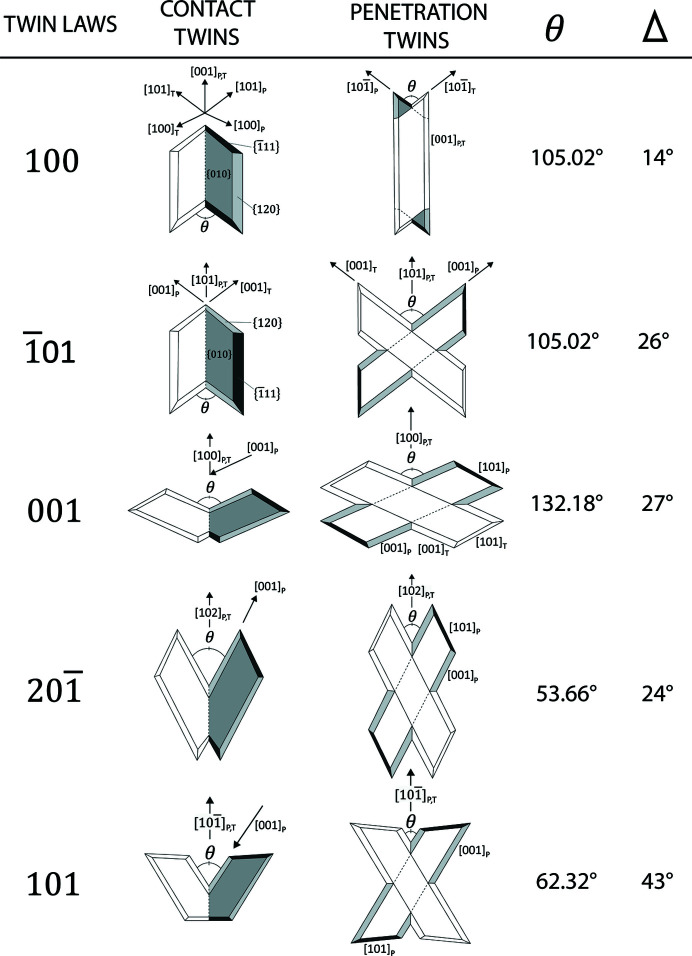
Geometry of contact and penetration twins, viewed along the [010] direction of gypsum. Modified from the work of Rubbo *et al.* (2012*a* ▸,*b* ▸) with permission from the American Chemical Society. Twin laws: 100, 101, 001, 201 and 101. For each twin law, the re-entrant angle value (θ) and the optical extinction angle value (Δ) have been reported. The extinction angles (Δ) were measured having adopted the structure defined by De Jong & Bouman (1939 ▸). The steps followed to measure the extinction angles are reported in Figs. S2 and S3 of the supporting information. Subscripts ‘P’ (parent) and ‘T’ (twinned) identify the two individuals that make up the twin.

Five different twin laws are possible for the gypsum structure (Follner *et al.*, 2002 ▸), and each twin law is described by a contact and penetration twin (Rubbo *et al.*, 2012*a* ▸,*b* ▸). Thus, at least ten different twin habits are related to gypsum (Fig. 1 ▸).

Geometrically, each twin law is characterized by a specific re-entrant angle (Fig. 1 ▸). By measuring its value, we can identify the twin law. However, the 100 and 101 twin laws have the same re-entrant angle (*i.e.* 105.02°). Thus, goniometry cannot distinguish these twins, and the formal way to correctly identify the 100 and 101 twin laws requires the measurement of the extinction angle (Δ) formed between the two individuals, by means of optical microscopy with crossed polarizers. This angle is 14 and 26° for the 100 and 101 twin laws, respectively. In addition, 100 and 101 penetration twins are recognized by their different habits: 100 penetration twins are more acicular than 101 penetration twins (Fig. 1 ▸).

Finally, a re-entrant angle and an arrowhead at opposite sides indicate a contact twin (Rubbo *et al.*, 2012*a* ▸), while two re-entrant angles observed at the opposite twin sides identify a penetration twin (Rubbo *et al.*, 2012*a* ▸,*b* ▸).

**Figure 2:**
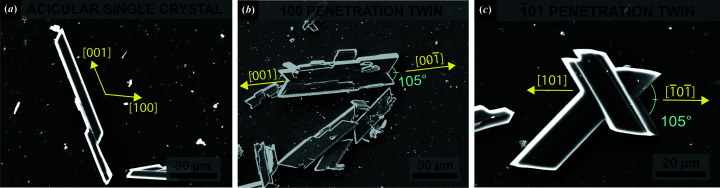
Gypsum crystals precipitated from G1 solution. (*a*) Acicular gypsum single crystals. (*b*) 100 penetration twin. (*c*) 101 penetration twin.

From the G1 solution (saturated in CaSO_4_·2H_2_O) we obtained the precipitation of (i) acicular single crystals elongated along [001] [Fig. 2 ▸(*a*)], (ii) 100 penetration twins with the two re-entrant angles developing along [001] and [001] [Fig. 2 ▸(*b*)], and (iii) 101 penetration twins with the two re-entrant angles developing along [101] and [101] [Fig. 2 ▸(*c*)].

**Figure 3:**
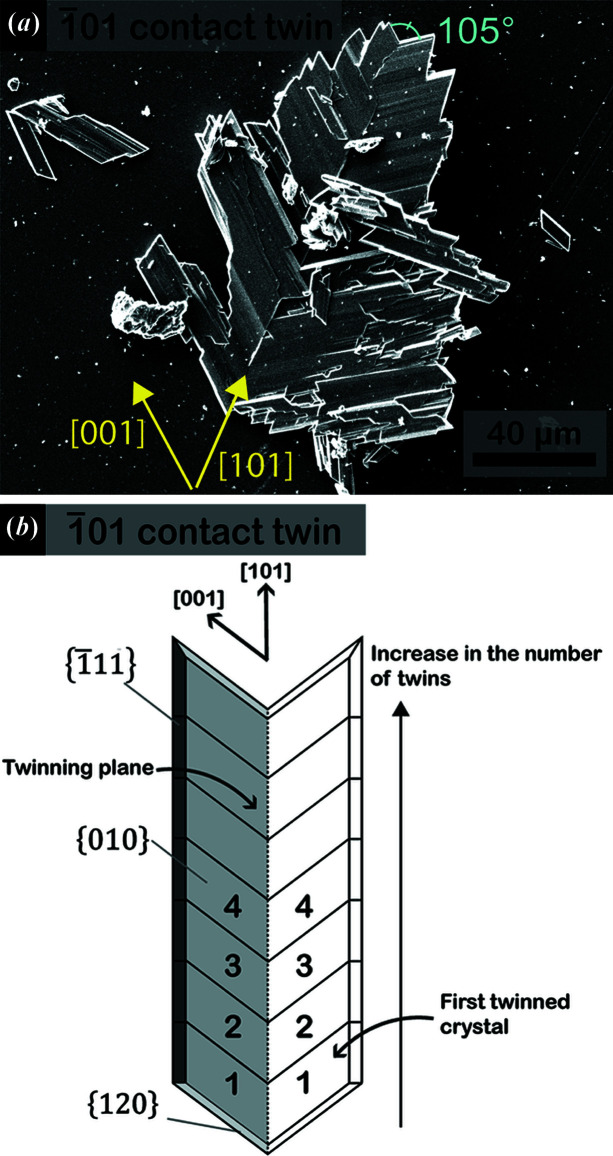
(*a*) 101 gypsum contact twin precipitated from a solution saturated in Ca-carbonate and calcium sulfate dihydrate (G2 solution). (*b*) Schematic representation of the 101 gypsum contact twins obtained in G2 solution.

From the G2 solution (saturated in CaSO_4_·2H_2_O and CaCO_3_) we precipitated acicular crystals, 100 penetration twins and 101 penetration twins (described above for the G1 solution), as well as a new twin morphology. Fig. 3 ▸(*a*) shows a twin characterized by a re-entrant angle of 105° and an optical extinction angle of 26° measured by means of optical microscopy (see Fig. S1 of the supporting information for the optical microscopy image). Moreover, a re-entrant angle at one side and an arrowhead at the opposite twin side are clearly observable. These features identify the 101 contact twin law.

Both 100 and 101 twin laws show the same re-entrant angle value. However, the sub-crystals composing 100 contact twins grow parallel to the twin plane (Otálora & García-Ruiz, 2014 ▸), whereas in 101 contact twins the main elongation of sub-crystals is oriented obliquely with respect to the twin plane (Fig. 3 ▸). Therefore, we propose that the main elongation of the sub-crystals forming the twin with respect to the twin plane is a useful tool to distinguish between 100 and 101 contact twins, especially for natural samples whose optical extinction angles can often be difficult to measure.

Moreover, the multiplicity of the gypsum twin operations is 2 (rotation of 180°, mirror plane or inversion center) and thus each twin law is composed of only two sub-crystals. In contrast, the experimentally obtained 101 contact twins show many sub-crystals that form the twin [Fig. 3 ▸(*a*)]. To explain this intriguing habit, a plausible mechanism might be that when the first 101 contact twin is formed – composed of only two sub-crystals – its re-entrant angle is the most reactive site, and thus a new 101 contact twin can statistically nucleate and grow in this position. When this mechanism is repeated several times, a multi-laminated twin is generated, as shown in Fig. 3 ▸. Explaining how this complex twin grows is beyond the scope of this work and will be investigated in future contributions.

### 3.2. The role of CO_3_^2−^ ions in the formation of the 101 gypsum contact twin law

Rapidcreekite is a rare hydrated Ca-sulfate–Ca-carbonate compound (Ca_2_SO_4_CO_3_·4H_2_O) found in association with gypsum and other carbonate minerals (Bots, 2011 ▸; Avdontceva *et al.*, 2021 ▸). It is composed of layers of Ca–SO_4_–Ca – CO_3_ with each Ca site coordinated by CO_3_, SO_4_ and two H_2_O groups (Cooper & Hawthorne, 1996 ▸). It belongs to the orthorhombic *Pbcn* space group and its unit-cell parameters are *a*_0_ = 15.49 Å, *b*_0_ = 19.18 Å, *c*_0_ = 6.15 Å, α = β = γ = 90° (Cooper & Hawthorne, 1996 ▸; Roberts *et al.*, 1986 ▸; Avdontceva *et al.*, 2021 ▸).

Due to the chemical and structural affinities between gypsum and rapidcreekite structures, Cooper & Hawthorne (1996 ▸) suggested a new formation mechanism for gypsum twins. They realized that the replacement of half the sulfate groups in gypsum produces the formula of rapidcreekite.

**Figure 4:**
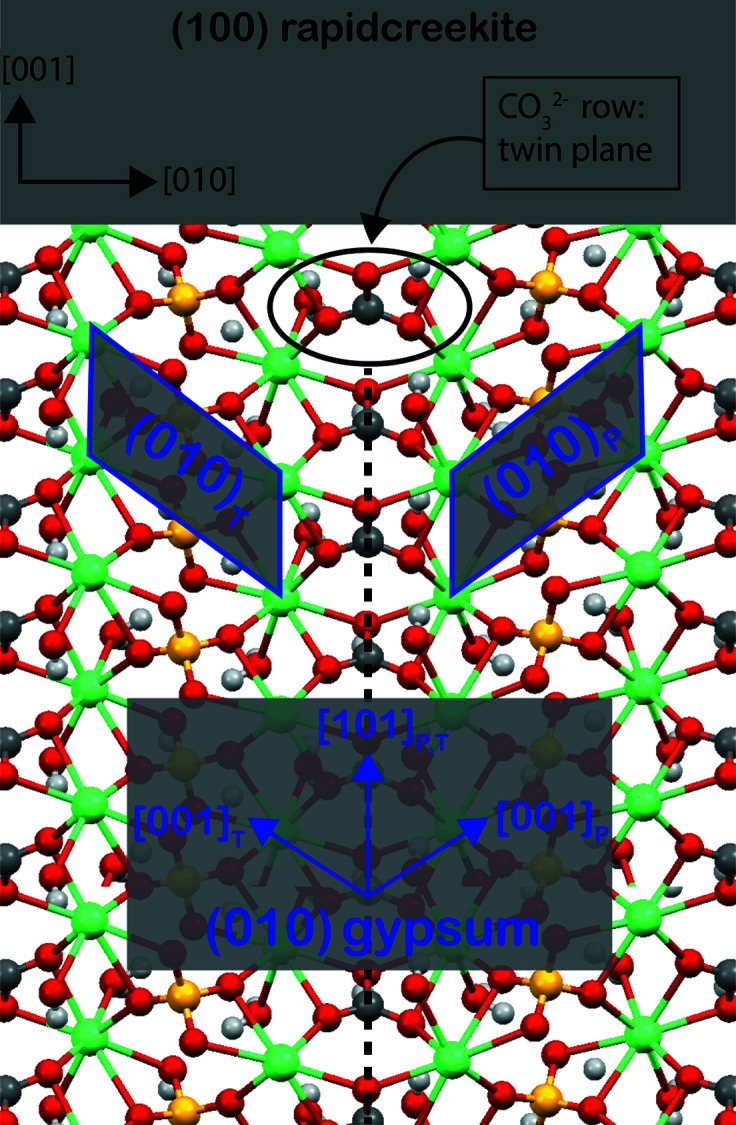
Projected structure of rapidcreekite perpendicular to the (100) plane: evidence that twinning in the gypsum structure can be attributed to the presence of rows of carbonate groups (black dashed line). Green – calcium; yellow –sulfur; red – oxygen; dark gray – carbon; light gray – hydrogen.

The resulting structure contains alternating sulfate and carbonate layers, and the sulfate groups in the alternate layers are rotated (*i.e.* twinned) by 180° with respect to the previous one (Fig. 4 ▸). Interestingly, the twin operation (*i.e.* 180° rotation) occurs along the [101] direction in our gypsum indexing, matching with the growth direction of the re-entrant angle in 101 gypsum twins.

Moreover, the effects of carbonate species in solution on gypsum crystallization were studied by Bots (2011 ▸) through *in situ* time-resolved crystallization experiments using wide-angle synchrotron X-ray scattering (WAXS) analyses [I22 beamline (SAXS/WAXS) at Diamond Light Source]. Bots observed that rapidcreekite is an intermediate product in gypsum formation.

Such results agree with our experimental observations involving the precipitation of 101 contact twins in carbonate-rich environments.

We investigated the 2D lattice coincidences between the {100} form of rapidcreekite and the {010} form of gypsum (Table 1 ▸). Both calculated linear and area misfits satisfy the constraints required for epitaxy interaction (*i.e.* linear and area misfit <14%) (Mutaftschiev, 2001 ▸), allowing us to consider rapidcreekite as a precursor in gypsum precipitation via an epitaxial mechanism, promoting the formation of the 101 contact twin law. Indeed, epitaxial growth is an acknowledged phenomenon responsible for triggering different growth morphologies (Kellermeier *et al.*, 2012 ▸; Bruno *et al.*, 2022*a* ▸) and polymorphs even if metastable phases are involved (Bruno *et al.*, 2022*b* ▸). The host phase forms a nanometre-thick intermediate structure whose faces act as the epitaxy substrate of the stable crystal, as reported in previous research where epitaxial relationships between two phases were investigated (Bruno *et al.*, 2022*a* ▸,*b* ▸).

### 3.3. Mineralogical implications

**Figure 5:**
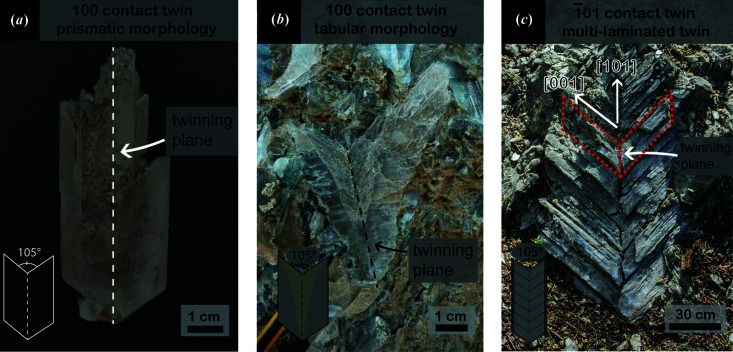
Examples of natural gypsum contact twins in modern and ancient evaporitic environments. (*a*) Centimetre-sized gypsum twin from the Atacama Desert, Chile. (*b*) Centimetre-sized Messinian selenitic gypsum from Piedmont basin, Italy (photograph courtesy of Marcello Natalicchio). (*c*) Metre-sized Messinian selenitic gypsum from ‘Vena del Gesso Romagnola’, Italy, composed of many sub-crystals obliquely elongated with respect to the twin plane (photograph courtesy of Piero Lucci).

The marine and lacustrine waters from which gypsum precipitates are rich in carbonate in its different species. Therefore, it is relevant to explore whether the 101 gypsum contact twins can be observed in these evaporitic environments. Gypsum crystals occur in evaporitic environments with three different contact twin habits:

(1) Prismatic habit [Fig. 5 ▸(*a*)] (Reid *et al.*, 2021 ▸) with a re-entrant angle value ranging between 100 and 105° and sub-crystals parallel to the twin plane. These features identify the 100 twin law.

(2) Tabular habit [Fig. 5 ▸(*b*)] (Natalicchio *et al.*, 2021 ▸; Costanzo *et al.*, 2019 ▸) with a re-entrant angle value ranging between 100 and 105° and sub-crystals parallel to the twin plane (Bigi *et al.*, 2022 ▸). Thus, the crystal follows the 100 twin law as shown in Fig. 5 ▸(*a*), but is characterized by a different habit.

(3) Multi-laminated habit [Fig. 5 ▸(*c*)], commonly called ‘Christmas tree’ (Rodríguez-Aranda *et al.*, 1995 ▸). In Fig. 5 ▸(*c*) the re-entrant angle value range is 98–101°, closer to the re-entrant angle value of 105° related to the 100 and the 101 gypsum twin laws.

Five twin laws are allowed for the gypsum structure (Follner *et al.*, 2002 ▸), and consequently only five re-entrant angle values are possible. However, it is reasonable to assume that, in the natural environment, gypsum crystals may be subjected to physical processes which may result in a deviation from the theoretical value of the twin law re-entrant angle, as demonstrated by the natural gypsum twins shown in Fig. 5 ▸.

Interestingly, in Fig. 5 ▸(*c*) the habit is composed of many specular and bladed individuals obliquely elongated with respect to the twin plane, like the 101 contact twins detected in the G2 solution. The [001] direction (*c* axis) is parallel to the elongation of the sub-crystals composing the twin; the same crystallographic directions describing 101 contact twins detected in G2 are obtained. Therefore, we propose that the 101 gypsum contact twins observed in the crystallization experiments from solutions saturated in Ca-carbonate are present in geological evaporitic environments. The high carbonate content in the brine from which the evaporites precipitate could promote the formation of this gypsum habit.

### 3.4. Fluid inclusion directions in 101 contact twins

FIs are small droplets of fluid trapped in minerals during their growth from the fluid phase; hence, rapid crystal growth events can result in polyhedric, dendritic or irregular FIs (Roedder, 1984 ▸; Bodnar *et al.*, 2003 ▸). However, after trapping, processes of recrystallization generally termed ‘necking down’ start to reduce the high surface energy of the FIs, especially in soluble minerals (Roedder, 1984 ▸; Bodnar *et al.*, 1985 ▸; Vityk *et al.*, 2000 ▸). The final result of such a necking down is the formation of FI morphologies reflecting those of the host mineral (Goldstein & Reynolds, 1994 ▸) at equilibrium with its mother solution, *i.e.* they represent the negative equilibrium shape (ES) of the host crystal.

**Figure 6:**
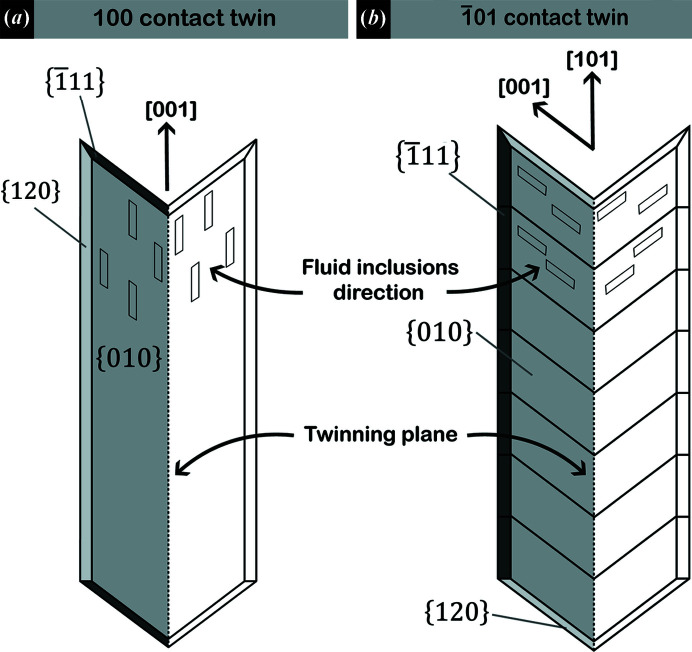
(*a*) 100 twin law, with FIs parallel to the twin plane. (*b*) 101 twin law, where inclusions are always oriented parallel to the [001] direction of single crystals, but obliquely with respect to the twin plane.

**Figure 7:**
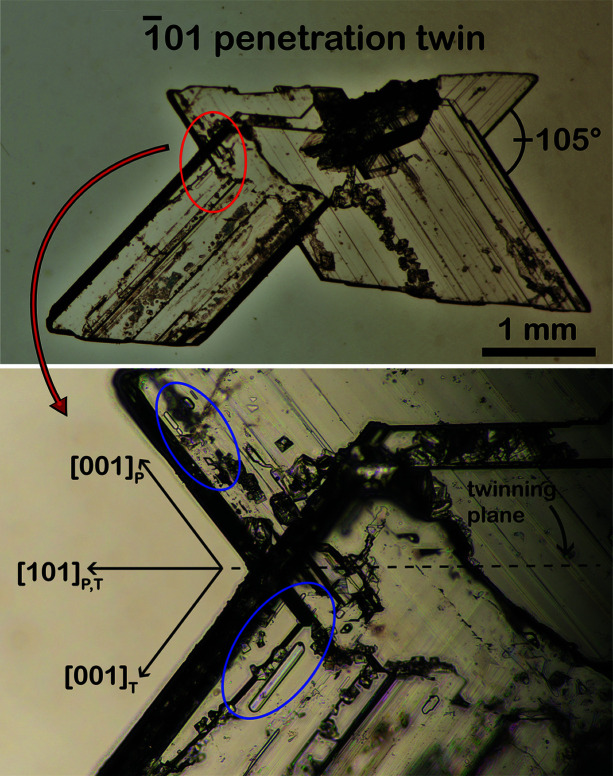
FIs in 101 twins are elongated along [001] and oriented obliquely with respect to the twin plane.

In the 100 gypsum twins, where the two individuals that form the twin grow along the [001] direction (Otálora & García-Ruiz, 2014 ▸; Costanzo *et al.*, 2019 ▸), the primary FIs show a negative ES elongated along the [001] direction parallel to the twin plane (Goldstein & Reynolds, 1994 ▸; Bigi *et al.*, 2022 ▸) [Fig. 6 ▸(*a*)]. In contrast, in 101 twins the [001] direction is oriented obliquely with respect to the twin plane, and thus, negative ES of FIs should develop obliquely with respect to the twin plane as well [Fig. 6 ▸(*b*)]. To test this hypothesis, optical microscopy was employed to observe FIs in millimetre-sized gypsum crystals belonging to the 101 twin law (Fig. 7 ▸). The crystal shown in Fig. 7 ▸ is one of those synthesized by Krüger *et al.* (2013 ▸).

Fig. 7 ▸ shows FIs grown along the [001] direction, oriented obliquely with respect to the twin plane and the growth direction of the re-entrant angle. Thus, the different orientations of the primary FIs’ negative ES with respect to the twin plane are a useful tool to distinguish between the 100 and 101 twin laws.

## 4. Conclusions

In this work, the effect of Ca-carbonate on the gypsum habit was studied by carrying out temperature-controlled laboratory experiments. Starting from two aqueous solutions saturated at 40°C in CaSO_4_·2H_2_O (G1) and in CaSO_4_·2H_2_O–CaCO_3_ (G2), we obtained gypsum by decreasing the temperature from 40 to 4°C.

We observed that Ca-carbonate triggers the formation of 101 gypsum contact twins. An epitaxial mechanism between the (100) face of rapidcreekite (Ca_2_SO_4_CO_3_·4H_2_O) and the (010) face of gypsum (CaSO_4_·2H_2_O) is suggested to explain the 101 gypsum contact twin formation. Both the atomic and the energetic levels of this mechanism will be discussed in detail in a forthcoming paper. Furthermore, when comparing the different gypsum twin morphologies observed in evaporitic environments with gypsum twins obtained in our experiments, we suggest that the occurrence of 101 gypsum contact twins in nature is probably more common than considered, and it could be an indicator of a high carbonate concentration in the brine from which they precipitate. Finally, it has been shown that the different orientations of primary FIs’ negative ES with respect to the twin plane and the main elongation of the sub-crystals that make up the twin are a useful tool to distinguish between the 100 and 101 twin laws.

To summarize, our results should help others to make better use of the twin laws observed in gypsum in ancient sedimentary successions as a pr­oxy for the chemistry of the original brine.

## Supplementary Material

Supporting figures. DOI: 10.1107/S1600576723002674/ei5095sup1.pdf

## Figures and Tables

**Table 1:** 2D lattice coincidences between the {100} form of rapidcreekite and the monoclinic {010} form of gypsum

Rapidcreekite (100)	Gypsum (010)	Geometric misfit (%)
[012] = 22.78 Å	4 × [100] = 22.52 Å	−1.14
[001] = 6.15 Å	[101] = 6.52 Å	5.67
2D area = 118.23 Å^2^	2D area = 128.59 Å^2^	8.00
