# Supplementary material for: 101 contact twins in gypsum experimentally obtained from calcium carbonate enriched solutions: mineralogical implications for natural gypsum deposits
Source: J Appl Crystallogr. 2023 Apr 13;56(Pt 3):603–10. doi: 10.1107/S1600576723002674 (PMC10241055; doi:10.1107/S1600576723002674)
Supplement: Supplementary file 1 [file j-56-00603-sup1.pdf]

JOURNAL OF
APPLIED
CRYSTALLOGRAPHY

Volume 56 (2023)

Supporting information for article:

\bar 101 contact twins in gypsum experimentally obtained from calcium carbonate enriched solutions: mineralogical implications for natural gypsum deposits

Andrea Cotellucci, Fermín Otálora, Àngels Canals, Joaquin Criado-Reyes, Luca Pellegrino, Marco Bruno, Dino Aquilano, Juan Manuel Garcia-Ruiz, Francesco Dela Pierre and Linda Pastero

S1. Optical microscopy image

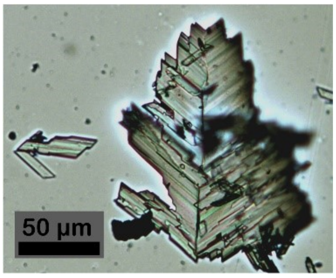

Figure S1 Optical microscopy image of $\bar{1}01$ contact twin observed in G2 solution. By means of optical microscopy under crossed polarizers we measured 26° as extinction angle among the individuals forming the twin, and thus, we identified the $\bar{1}01$ twin law.

S2. The extinction angles for the five twin laws

To calculate the extinction angles of the five gypsum twin laws, it is required to:

i) Project the optical indicatrix of gypsum on the (010) plane (Fig. S2).

ii) Apply the twin law to generate the “twinned optical indicatrix” (T) (Fig. S3).

iii) Measure the angle value generated between the “old” refractive indices (e.g., γ) and the new one (γ_T or α_T) (Fig. S3). The value of this angle represents the extinction angle for the twin law, i.e., how much to rotate the crystal to move the twinned sub-crystal into extinction position when the parent sub-crystal is already extinguished.

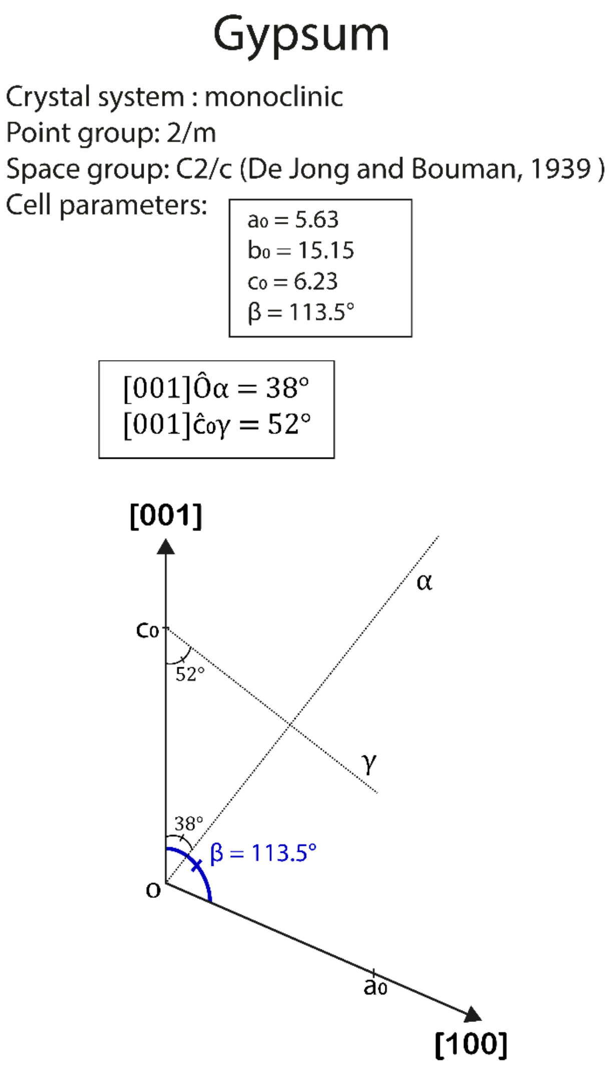

Figure S2 Gypsum reference frame used to measure the extinction angles of the five gypsum twin laws (De Jong and Bouman, 1939). α and γ represent the refractive indices of gypsum (α=1.519 – 1.521; γ=1.529 – 1.531) (Chang et al., 1996). The monoclinic *C2/c* space group of gypsum ($a_0 = 5.63$, $b_0 = 15.15$, $c_0 = 6.23$ Å; $\alpha = \gamma = 90°$; $\beta = 113.50°$) (De Jong and Bouman, 1939) was used to project the optical indicatrix of gypsum on the (010) plane.

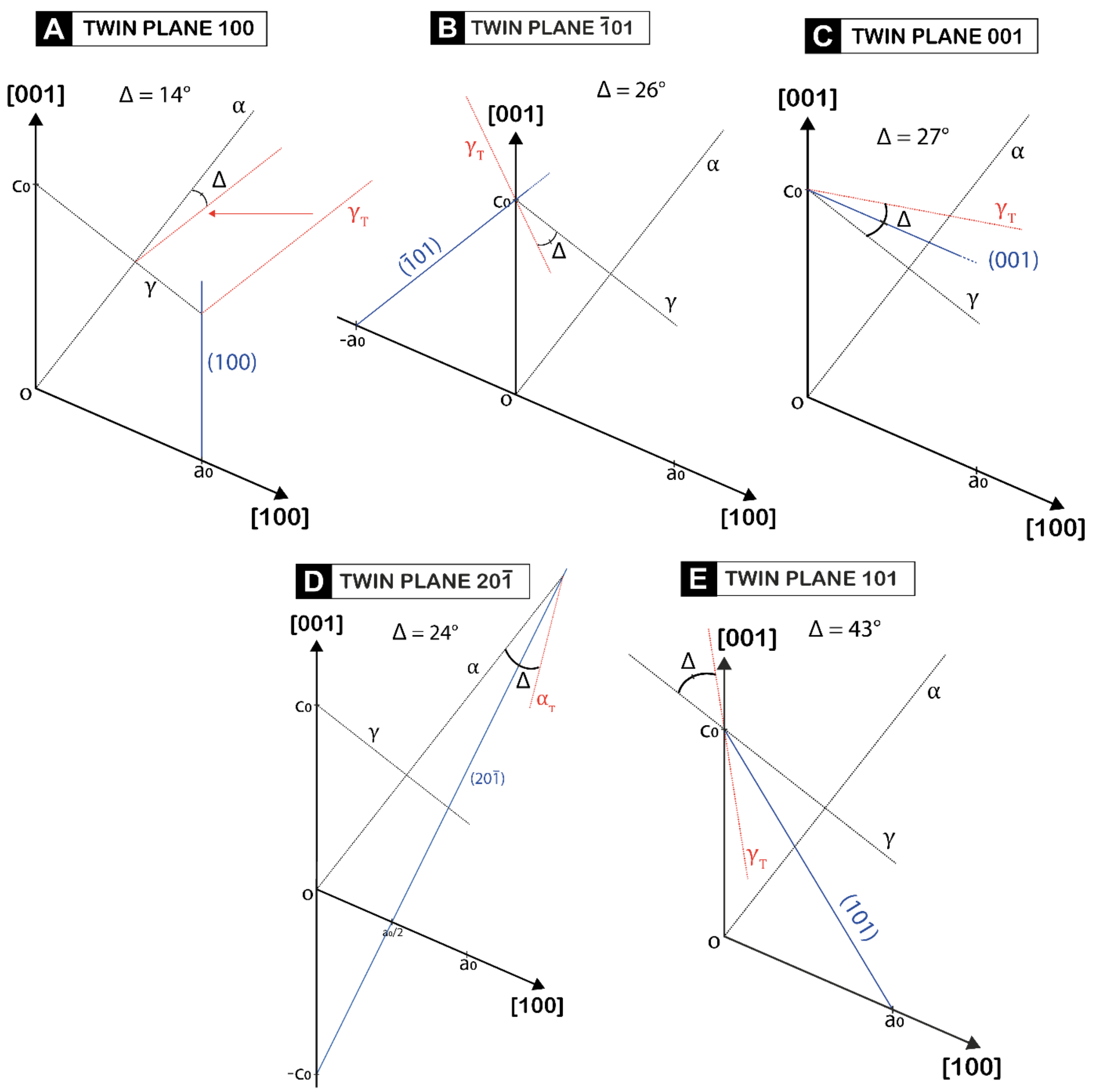

Figure S3 Extinction angles of the five gypsum twin laws.
